# Novel FLT3/AURK multikinase inhibitor is efficacious against sorafenib-refractory and sorafenib-resistant hepatocellular carcinoma

**DOI:** 10.1186/s12929-022-00788-0

**Published:** 2022-01-21

**Authors:** You-Liang Lai, Kai-Hung Wang, Hsing-Pang Hsieh, Wan-Ching Yen

**Affiliations:** 1 Institute of Biotechnology and Pharmaceutical Research, National Health Research Institutes, Zhunan Town, Maioli County, Taiwan; 2 Present Address: Department of Medical Research, Hualien Tzu Chi Hospital, Buddhist Tzu Chi Medical Foundation, Hualien, Taiwan

**Keywords:** Hepatocellular carcinoma, Multikinase inhibitors, Aurora kinases, Sorafenib resistance

## Abstract

**Background:**

Hepatocellular carcinoma (HCC) is the sixth most common type of cancer and has a high mortality rate worldwide. Sorafenib is the only systemic treatment demonstrating a statistically significant but modest overall survival benefit. We previously have identified the aurora kinases (AURKs) and FMS-like tyrosine kinase 3 (FLT3) multikinase inhibitor DBPR114 exhibiting broad spectrum anti-tumor effects in both leukemia and solid tumors. The purpose of this study was to evaluate the therapeutic potential of DBPR114 in the treatment of advanced HCC.

**Methods:**

Human HCC cell lines with histopathology/genetic background similar to human HCC tumors were used for in vitro and in vivo studies. Human umbilical vein endothelial cells (HUVEC) were used to evaluate the drug effect on endothelial tube formation. Western blotting, immunohistochemical staining, and mRNA sequencing were employed to investigate the mechanisms of drug action. Xenograft models of sorafenib-refractory and sorafenib-acquired resistant HCC were used to evaluate the tumor response to DBPR114.

**Results:**

DBPR114 was active against HCC tumor cell proliferation independent of p53 alteration status and tumor grade in vitro. DBPR114-mediated growth inhibition in HCC cells was associated with apoptosis induction, cell cycle arrest, and polyploidy formation. Further analysis indicated that DBPR114 reduced the phosphorylation levels of AURKs and its substrate histone H3. Moreover, the levels of several active-state receptor tyrosine kinases were downregulated by DBPR114, verifying the mechanisms of DBPR114 action as a multikinase inhibitor in HCC cells. DBPR114 also exhibited anti-angiogenic effect, as demonstrated by inhibiting tumor formation in HUVEC cells. In vivo, DBPR114 induced statistically significant tumor growth inhibition compared with the vehicle control in multiple HCC tumor xenograft models. Histologic analysis revealed that the DBPR114 treatment reduced cell proliferation, and induced apoptotic cell death and multinucleated cell formation. Consistent with the histological findings, gene expression analysis revealed that DBPR114-modulated genes were mostly related to the G2/M checkpoint and mitotic spindle assembly. DBPR114 was efficacious against sorafenib-intrinsic and -acquired resistant HCC tumors. Notably, DBPR114 significantly delayed posttreatment tumor regrowth and prolonged survival compared with the regorafenib group.

**Conclusion:**

Our results indicated that targeting AURK signaling could be a new effective molecular-targeted agent in the treatment of patients with HCC.

**Supplementary Information:**

The online version contains supplementary material available at 10.1186/s12929-022-00788-0.

## Background

Liver cancer is the sixth most common cancer and fourth most deadly cancer worldwide [1]. Hepatocellular carcinoma (HCC) accounts for 90% of primary liver cancer. Its incidence and mortality are geographically related, with Eastern Asia, South-Eastern Asia, and Western Africa exhibiting a high incidence rate. The prognosis is poor; median survival times are typically less than 1 year, and overall survival (OS) rates less than 5% [2]. Although early-stage HCC can be treated with surgical liver resection or liver transplantation, the 5-year postsurgical recurrence rate can reach 70% [3]. A primary reason for the poor prognosis in patients with HCC is the absence of potent therapies, particularly in the advanced stage. Conventional chemotherapy (single agent or combination) is not routinely used for advanced HCC because this cancer is chemorefractory and chemotherapeutic agents induce adverse events [3]. Poor hepatic reserves increase the difficulty of managing HCC clinically [3].

The pathogenesis of HCC is highly complex. Several key signal transduction pathways have been implicated in HCC pathogenesis, including those mediated by epidermal growth factor (EGF)/EGF receptor (EGFR), vascular endothelial growth factor (VEGF)/VEGF receptor (VEGFR), platelet-derived growth factor (PDGF)/PDGF receptor (PDGFR), hepatocyte growth factor (HGF)/mesenchymal–epithelial transition factor (MET) receptor, insulin-like growth factor (IGF)/IGF receptor (IGFR); RAS/RAF/mitogen-activated protein kinase kinase (MEK)/extracellular signal-regulated kinase (ERK), phosphatidylinositol-3-kinase (PI3K); phosphatase and tensin homologue deleted on chromosome ten (PTEN)/protein kinase B (AKT)/mammalian target of rapamycin (mTOR) signaling pathways, and Wnt/β-catenin [4]. In the last two decades, several molecular-targeted agents have been developed and tested. Notably, targeted agents that inhibit angiogenesis factors while simultaneously inhibiting other key proangiogenic factors in HCC, such as fibroblast growth factor receptor (FGFR) and MET signaling, have provided insight into the underlying pathogenesis of HCC tumors (see reviews in [5]). To date, sorafenib (Nexavar), a multikinase inhibitor targeting RAF serine/threonine kinases, and VEGFR1-3, PDGFR beta (PDGFRβ), and RAS/RAF/MEK/ERK signaling pathways, is the first approved molecular-targeted agent that demonstrated survival benefits in patients with advanced HCC in a 2007 study [6]. Since then, sorafenib has remained the standard of care for first-line systemic therapy in advanced HCC with preserved liver function. However, the response duration is short, and medication prescription is often discontinued as a result of intolerable side effects or drug resistance. In the past 10 years, attempts to develop more potent first-line agents to replace sorafenib and to identify potent second-line agents after disease progression under sorafenib treatment have been unsuccessful (see reviews in [5]). In 2018, lenvatinib (Lenvima), a multikinase inhibitor targeting VEGFR1-3, FGFR1-4, PDGF alpha (PDGFRα), rearranged during transfection receptor (RET) proto-oncogene receptor tyrosine kinase, and KIT proto-oncogene receptor tyrosine kinase (KIT), was approved by European and Asian food and drug administration (FDA) authorities as an alternative first-line agent for the treatment of patients with unresectable HCC. This decision was based on the results of a multicenter, randomized, open-label, phase 3 trial comparing the efficacy and safety of lenvatinib to that of sorafenib in first-line treatment of patients with unresectable HCC (NCT01761266), which demonstrated statistically significant and clinically meaningful improvements in terms of progression-free survival (PFS), time to progression (TTP), and overall response rate with improved safety profiles; however, the OS of lenvatinib is similar to that of sorafenib [7]. Regorafenib (Stivarga), a structurally unique multikinase inhibitor targeting several cancer-associated kinases, including angiogenic (VEGFR1-3, tunica interna endothelial-2 (TIE-2)), stromal (PDGFRβ and FGFR), and oncogenic receptor tyrosine kinases (KIT, RET, and RAF) [8, 9], was approved by the US FDA in April 2017 as a second-line treatment for patients who fail to respond to first-line sorafenib therapy [10]. Another second-line agent cabozantinib, a multikinase inhibitor targeting VEGFR1-3, MET, AXL receptor tyrosine kinase, KIT, and RET, was approved by the US FDA in January 2019 for patients with HCC who have been previously treated with sorafenib. This decision was based on the positive results of the worldwide randomized, placebo-controlled, phase 3 trials in patients with unresectable HCC who received one or two prior lines of treatment including sorafenib (NCT01908426) [11]. Sorafenib, regorafenib, lenvatinib, and cabozantinib share similar mode of actions in that they are multikinase inhibitors that block protein kinases involved in tumor angiogenesis (VEGFRs), oncogenesis (RAS, RAF, KIT, and RET), and metastasis (PDGFR). Regorafenib has more potent cytotoxicity and favorable side effect profiles but is unsuitable for patients intolerant to sorafenib because of their similar modes of action (see reviews in [5]); for cabozantinib, dose reduction is a frequent concern [12]. Thus, the development of targeted agents with novel mechanisms of action is essential for the treatment of the subset of patients with HCC insensitive to the aforementioned agents or who have relapsed from these therapies.

Aurora kinase (AURK) isoforms A, B, and C (AURKA, AURKB, and AURKC) are members of the serine/threonine kinase family and are involved in the regulation of various stages of mitosis. Both AURKA and AURKB are essential during mitosis, whereas AURKC plays a crucial role in spermatogenesis [13]. Aberrant expression of AURKA and AURKB has been reported in both solid tumors and hematologic malignancies, including several forms of leukemia and cancer of the breast, colon, lung, pancreas, prostate, and thyroid (see reviews in [14]). In HCC, overexpression of AURKA and AURKB is associated with tumor aggressiveness, an unfavorable prognosis, and poorer outcomes [15–17], and their co-expression is an independent predictor of PFS and OS [18]. Preclinical studies have demonstrated that the pharmacologic inhibition of AURKs or knockdown of *AURK* reduced HCC tumor cell growth, suppressed cell invasion, and induced cell cycle arrest and apoptosis in vitro and in vivo [19–22]. In addition to its role in malignant transformation and cancer development, AURKA expression has been linked to treatment resistance in various solid tumors including HCC (see reviews in [14]). Zhang et al. [23] have reported that silencing AURKA enhanced the sensitivity of HCC cells to chemotherapeutic agents doxorubicin and cisplatin, whereas AURKA overexpression reduced the HCC human cellular response to chemotherapy-induced apoptosis. Further investigation into the mechanisms of AURKA-mediated chemoresistance revealed that AURKA enhanced nuclear factor-kappa B (NF-κB) activity and promoted microRNA-21 transcription, which downregulated phosphatase and PTEN and inhibited caspase-3-mediated apoptosis induction [23]. Similar mechanisms were also involved in AURKA-mediated radioresistance, with Shen et al. [24] revealing that upregulation of AURKA reduced radiotherapy-induced apoptosis in human HCC cells through the activation of NF-κB signaling, and that knockdown of AURKA resensitized radioresistant HCC cells to radiotherapy. These findings indicated that targeting the AURKA/NF-κB signaling pathway could be a therapeutic strategy to overcome chemoresistance and radioresistance in HCC.

The tumor suppressor p53 protein constitutes one of the most frequently altered genes in HCC; p53 alteration is positively correlated with AURKA and AURKB [15, 16]. Dauch et al. [25] have demonstrated that in p53-altered HCC, AURKA formed a complex with MYC protein to promote MYC-mediated cell cycle re-entry and tumor cell survival. Interruption of AURKA and MYC interaction through the conformation-changing AURKA inhibitors MLN8237 and CD532 prevented AURKA–MYC complex formation, resulting in MYC degradation and cell death. Mice bearing *TP53*-variant or *TP53*-deleted human HCC tumors were hypersensitive to conformation-changing AURKA inhibitor-mediated tumor growth [25]. These data indicated that agents that interfere with AURKA and MYC interaction could be a therapeutic strategy for the treatment of patients with p53-variant HCC.

We previously have reported the development of a dual FMS-like tyrosine kinase 3 (FLT3)/AURK multikinase inhibitor DBPR114, also known as BPR1K871 [26]. Unlike sorafenib, regorafenib, and lenvatinib, which are designed to target kinase pathways involved in angiogenesis (VEGFRs) and RAS/RAF/MEK/ERK oncogenic pathways, DBPR114 primarily targets oncogenic receptor kinase FLT3/AURK/KIT/RET signaling pathways. DBPR114 was initially developed as a dual FLT3/AURK multikinase inhibitor for the treatment of FLT3 internal tandem duplication alteration-positive acute myeloid leukemia (AML) tumors and FLT3 wild-type AML tumors. DBPR114 potently inhibited the growth of FLT3-variant AML cells but was minimally effective against FLT3-negative leukemia cell lines [26]. The concentration required to produce half-maximal growth inhibition, IC_50_, in FLT3-expressing AML cells through DBPR114 introduction was tenfold greater than that of the two known AURK inhibitors VX680 and barasertib [26]. In addition to AML, DBPR114 also exhibited a broad spectrum of antitumor activity against various solid tumor type cancers of colon, stomach, lung, and pancreas as well as uterine sarcoma, and induced significant tumor volume reduction in colon and pancreatic xenograft tumor models [26]. Mechanistic studies of MV4-11 leukemia cells and HCT-116 colon cancer cells revealed that DBPR114 modulated FLT3 and AURKA/B targets inside the cells and induced the accumulation of multinucleated cells, which indicates mitotic checkpoint override through AURKB inhibition [26]. These findings prompted us to examine the use of DBPR114 as a multikinase inhibitor in the treatment of patients with advanced HCC. We utilized six human HCC cell lines with histopathology/genetic background similar to those of human HCC tumors [27] to evaluate the therapeutic potential of this compound. The DBPR114-mediated drug effect was determined at the cellular and molecular level to identify potential pharmacodynamic biomarkers for monitoring target engagement and drug response.

## Methods

### Cell lines and reagents

All human HCC cell lines and HUVEC were obtained from American Type Culture Collection (Rockville, MD, USA). Huh1, Huh7, PLC/PRF/5, Hep3B, HA22T/VGH, and HA59T/VGH cells were cultured in Dulbecco's Modified Eagle Medium (DMEM), and human umbilical vein endothelial cells (HUVEC) were cultured in DMEM and F-12K medium (50%: 50%, v:v). All culture media were supplemented with 10% fetal bovine serum (FBS) and were maintained in a humidified atmosphere of 5% CO_2_ at 37 °C. Cell culture media and supplements were obtained from Hyclone (Thermo Fisher Scientific, Waltham, MA, USA). The HCC cell lines and their histopathology are listed in Table 1. DBPR114 was synthesized at Institute of Biotechnology and Pharmaceutical Research, National Health Research Institutes, Taiwan. Sorafenib and regorafenib were purchased from BOC Sciences (Shirley, NY, USA), and VX680 and nocodazole from Abcam (Cambridge, MA, USA). Sorafenib and DBPR114 were dissolved in 100% dimethyl sulfoxide (DMSO; Sigma-Aldrich, St. Louis, MO, USA) and diluted with culture medium to the desired concentration, resulting in a final DMSO concentration of 0.1% for the in vitro studies; the solvent control contained 0.1% DMSO in culture medium only.

**Table 1:** Antiproliferative activity of DBPR114 in human HCC cell lines in vitro

Cell line	Histopathology	Growth inhibition, IC_50_ (μM)	
		DBPR114	Sorafenib
HA22T/VGH	HCC, p53^MT^, poorly differentiated, HBV^+^/HCV^−^	0.7	8.6
HA59T/VGH	HCC, p53^MT^, poorly differentiated, HBV^+^/HCV^−^	1.7	8.3
Huh1	HCC, p53^MT^, moderately differentiated, HBV^−^/HCV^−^	1.9	9.5
Huh7	HCC, p53^MT^, moderately differentiated, HBV^−^/HCV^−^	1.7	8.4
PLC/PRF/5	HCC, p53^MT^, moderately differentiated, HBV^+^/HCV^−^	2.1	6.5
Hep3B	HCC, p53^null^, well differentiated, HBV^+^/HCV^−^	1.5	6.6

HCC cells were treated with DBPR114 or sorafenib at various concentrations for 72 h. Cell viability was assessed through WST-8 cell proliferation assay. IC_50_ values represent the mean of two independent experiments with eight concentrations and six replicates per concentration

p53^null^: p53 Deletion; p53^MT^ : Mutant-type p53; HBV^+^ : Hepatitis B-positive virus; HBV^−^: Hepatitis B-negative virus; HCV^− ^^-^: Hepatitis C-negative virus

### Cell proliferation assay

HCC cells were plated in 96-well microtiter plates, with 3000 cells per well, and incubated in 10% FBS-containing cell culture medium overnight at 37 °C. Cells were then treated with the vehicle or various concentrations of the compound in medium for 72 h. Viable cells were quantified using the WST-8 cell proliferation assay kit (Cayman Chemical, Ann Arbor, MI, USA) according to the manufacturer’s recommended protocol. Results were determined through measurement of the absorbance at 490 nm using a Perkin Elmer Wallac 1420 VICTOR2 microplate reader (Shelton, CT, USA). The IC_50_ value was defined as the compound concentration that induced a 50% reduction in cell viability in comparison with the DMSO-treated (vehicle) control; this was calculated using GraphPad Prism 5 (GraphPad Software Inc., San Diego, CA, USA).

### Apoptotic cell death detection assay

At 10,000 cells per well, cells were seeded in 96-well plates overnight. The cells were then treated with DBPR114 at various doses or 0.1% DMSO for 48 h in culture medium. Apoptotic cell death was measured using a DNA fragmentation kit according to manufacturer’s instruction (Cell Death Detection enzyme-linked immunosorbent assay (ELISA) PLUS, Roche, Mannheim, Germany). In brief, cytoplasmic fractions of the control and treated cells were transferred into streptavidin-coated 96-well plates and incubated with biotinylated mouse anti-histone antibody and peroxidase-conjugated mouse anti-DNA antibody at room temperature for 2 h. Absorbance was determined at 405 to 490 nm using a Perkin Elmer Wallac 1420 VICTOR2 microplate reader.

### Endothelial tube formation assay

Matrigel (BD Biosciences, San Jose, CA, USA) matrix was thawed and maintained on ice at 4 °C; 50 μL of the matrix was transferred to each of the 96-microwell culture plates. The plates were incubated at 37 °C for at least 1 h to allow the matrix solution to solidify before treatment. Aliquots of 200 μL of HUVEC suspended in endothelial cell growth medium with 2.5% FBS (1.5 × 10^4^ cells/well) were placed in the 96-well Matrigel-coated plates (Corning, Glendale, AZ, USA). The vehicle control and DBPR114 were then added to each well in triplicate and incubated at 37 °C for 18 h. Following incubation, the morphology of the endothelial cell tubes in the individual wells was evaluated through photomicroscopy (Olympus CK40 microscope, Tokyo, Japan). The failure of the formation of continuous networks between cell bodies in the presence of drug treatment was photographed and scored depending on the extent of tube disruption at a magnification of × 40. The total tube length in each picture was measured, with ≥ 30% tube formation inhibition relative to the vehicle-treated control group indicating significant anti-angiogenic activity.

### Flow cytometry cell cycle analysis

To evaluate the effect of DBPR114 on cell cycle distribution, cells were seeded in a six-well plate with 10^5^ cells per well and treated with DBPR114 in the presence of 10% FBS-containing cell culture medium for 48 h. Thereafter, cells were harvested through trypsinization and washed with phosphate buffered saline (PBS). Cells were fixed in ice-cold 70% ethanol, washed, resuspended in PBS, treated with ribonuclease, and then stained with propidium iodide. Cell cycle distribution was assessed with a BD Biosciences FACSCalibur flow cytometry system and quantified using FlowJo software (BD Biosciences).

### Immunoblot analysis

To determine the effect of DBPR114 on the inhibition of AURK and its substrate histone H3, HCC cells were incubated in 10% FBS-containing cell culture medium for 16 h with 40 ng/mL nocodazole and then underwent drug treatment for 2 h at various concentrations. The AURK inhibitor V680 was used as a positive control for the detection of AURK and its substrate histone H3 proteins. For analyses of MET and its downstream effector molecules, HCC cells were treated with DBPR114 at the indicated concentrations for 2 h. Twenty-five ng/mL HGF (ligand for c-MET) was added 10 min prior to the end of drug treatment. For the AXL and MERTK analysis, HCC cells were incubated with the vehicle control or DBPR114 in the presence of 200 ng/mL GAS6 (ligand for AXL and MERTK) for 30 min. The cell lysates were prepared and analyzed using Western immunoblotting. The sources of primary antibodies were as follows: anti-phospho-AURKA (Thr288), anti-phospho-AURKB (Thr232), anti-phospho-ERK (Thr202/Tyr204), anti-phospho-MEK (Ser217/221), anti-phospho-AKT (Ser473), anti-phospho-MET (Tyr1234/1235), anti-phospho-AXL (Tyr702), anti-ERK1/2, anti-MEK1/2, anti-MET, and anti-AXL (C89E7) were procured from Cell Signaling Technology; anti-phospho-MER proto-oncogene tyrosine kinase (MERTK) (Y749 + Y753 + Y754), anti-MERTK (Y323), anti-AURKA, anti-AURKB, anti-phosphorylated histone H3 (Ser10) and anti-histone H3 from Abcam. Anti-glyceraldehyde 3-phosphate dehydrogenase (GAPDH) was also procured from Abcam, and anti-AKT/PKBα (AW24) from MilliporeSigma (Burlington, MA, USA). The secondary antibody horseradish peroxidase (HRP)-linked goat anti-rabbit IgG (111-035-003) was purchased from Cell Signaling Technology, and the recombinant human HGF and Gas6 from Abcam. Autoradiographs were scanned for densitometric analysis using Image J software (http://imagej.nih.gov/ij, NIH, Bethesda, MA, USA).

### In vivo animal studies

All liver cancer cells were determined to be free of *Mycoplasma* spp. prior to their injection into animals. Nonobese diabetic, severe combined immunodeficiency (NOD/SCID) mice (BioLASCO, Taipei, Taiwan) were used for the in vivo experiments because of their higher tumor take and growth rate compared with that of athymic nude mice from the pilot study. Subcutaneous injections were made into the left flank region of the NOD/SCID mice with a 25-gauge 5/8-in needle (n = 7–12 animals per group). Treatment was initiated after randomization, with the inclusion of tumors 100 to 150 mm^3^ in size. The dosing vehicle for DBPR114 was prepared with 0.152 M lactic acid in double distilled water and diluted with 5% dextrose in water (D5W) at a ratio of 1:3. The pH value of the vehicle was adjusted with 1 N sodium hydroxide to 3.8 for animal dosing. The DBPR114 dosing solution was prepared through dilution of the DBPR114 stock solution (20 mg/mL in 0.152 M lactic acid) with 1.2 mL D5W, resulting in a 8-mg/mL dosing solution. DBPR114 was dosed at 40 mg/kg intravenously once a week. Both sorafenib and regorafenib were prepared daily through dissolution in a Cremophor EL (Sigma-Aldrich, St. Louis, MO, USA), ethanol, and 0.9% sodium chloride solution (12.5%:12.5%:75%, v:v:v), and dosed at 30 mg/kg orally once a day, 5 days per week. Treatment length varied depending on the individual tumor growth rate. The doses and dose regimens used for DBPR114, sorafenib, and regorafenib in this study were determined to be the maximal tolerated doses for a treatment duration up to 6 weeks in NOD/SCID mice. Tumor growth was measured using an electronic caliper, and volumes were calculated as L × W × W/2, where L and W are the length and width, respectively. Tumor size and animal body weight were measured once per week after tumor cell inoculation. Tumor response at the end of the study was calculated as tumor growth inhibition (TGI):(1 – T/C) × 100, where T and C represent the mean tumor volume (mm^3^) of the test and vehicle-treated group, respectively.

To determine the effect of DBPR114 as a second-line treatment for intrinsic sorafenib-resistant tumors, sorafenib insensitive HA22T/VGH tumor-bearing mice were treated with sorafenib, DBPR114, or regorafenib for 6 weeks. Treatment was discontinued thereafter, and the animals were monitored for progressive tumor regrowth. To assess the antitumor activity of DBPR114 in tumors that developed sorafenib-acquired resistance, Huh7 tumor-bearing animals exhibiting sorafenib treatment-induced tumor growth, that is, tumors with a volume increase of ≥ 30% during treatment, were removed at the end of study. The remaining animals with sorafenib treatment-sensitive tumors, that is, tumors with a volume increase of < 30% or regressive tumors, were harvested and reimplanted into recipient mice. These animals were randomized and received sorafenib treatment for 4 weeks, during which the average tumor size reached 100 mm^3^. This process was repeated twice when tumors developed acquired resistance to sorafenib; that is, when tumors responded to sorafenib for at least 2 weeks but then exhibited a ≥ 30% increase in tumor volume within 5 days. Thereafter, sorafenib-resistant Huh7 tumors were harvested and implanted into recipient mice. Additional file 1: Fig. S1 illustrates the flow scheme of the in vivo sorafenib-acquired resistant tumor model. The mice bearing sorafenib-acquired resistant Huh7 tumors were randomized and treated with the vehicle control, sorafenib, DBPR114, or regorafenib for 25 days when the average tumor size reached 100 mm^3^. The treatment was then discontinued, and the animals were monitored for tumor growth and body weight changes. For the survival study, the animals were sacrificed when their tumor volume reached 2000 mm^3^, or when they exhibited a body weight loss > 10%. Kaplan–Meier survival curves were used at the study endpoint to calculate the percentage of animals remaining in the study in relation to the time scale. All experiments were conducted in accordance with the protocols approved by National Health Research Institutes’ Institutional Animal Care and Use Committee.

### mRNA sequencing and gene expression analysis

Three hours after the final dose, three to four representative tumor samples (tumor size within one standard deviation [SD] of the mean tumor volume) were harvested from the Huh7 tumor-bearing animals treated with vehicle control, DBPR114, and sorafenib. Nonnecrotic tissues were carefully removed from the tumors and immediately snap-frozen at − 80 °C until later use for gene and protein expression analysis; the remaining tissues were fixed in formalin for histologic evaluation. Drug treatment-induced changes in the target gene expression profiles and key pathway components in the tumors were examined through RNA sequencing analysis. For gene expression analysis, RNA was isolated from the snap-frozen xenograft tumor tissue using the RNeasy Fibrous Tissue Mini Kit (Qiagen, Germantown, MA, USA) with DNAse I treatment, as described in the manufacturer’s protocol. The quality of RNA was assessed with an Agilent Bioanalyzer. The sequencing libraries were prepared following the supplier’s protocols for sequencing mRNA samples (Illumina, Foster City, CA, USA). The FASTQ sequence reads were aligned using the human genome hg19 TopHat (v2.0.9) application with default parameters [28] and Bowtie (v1.0.0) [29]. Following alignment of the sequence reads, the uniquely mapped reads were counted for each gene by using the HTSeq Python script (v0.6.1) (https://pypi.python.org/pypi/HTSeq) [30]. The raw counts per gene in each xenograft tumor were then normalized as fragments per kilobase per million mapped reads to represent the expression level of the gene in the tumor. Differentially expressed genes were identified using the Bioconductor package DESeq2 (v1.10.1; DESeq2_1.10.1.tar.gz) [31]. A heatmap was generated through the color-coding of standardized log gene expression levels (mean, zero; SD, one). The RNA sequencing data were subjected to gene set enrichment analysis (GSEA; http://www.broadinstitute.org/gsea) to identify the enrichment or depletion of defined gene expression signatures in reference to the Molecular Signatures Database (MSigDB; www.braodinstitute.org.msigdb); the Bioconductor topGO pathway annotation software package (v3.0; https://bioconductor.org/packages/topGO/) was employed to map genes to their cellular components and regulatory networks. Genes differentially expressed between the control and treated samples were identified through Fisher’s exact test. The genes that were significantly regulated between the treatment and vehicle control were selected based on a false discovery rate (FDR) of < 0.05 and absolute fold change of ≥ 2 on pretransformed expression on a log2 scale. The *p* value was adjusted for multiple testing using the Benjamini–Hochberg procedure to estimate the FDR.

### Immunohistochemical analysis

Formalin-fixed, paraffin-embedded sections were dewaxed. Heat induced epitope retrieval was performed in a water bath containing citrate buffer (Dako North America, Carpinteria, CA, USA), blocked with 1% hydrogen peroxide and then treated for 30 min with CAS-Block (Invitrogen, Carlsbad, CA, USA) before primary antibody incubation. The primary antibodies were rabbit monoclonal anti-Ki-67 (1:200 dilution; Thermo Fisher Scientific) and rabbit monoclonal anti-CD31 (1:75 dilution; Abcam). Staining signals were detected using the Starr Trek Universal horseradish peroxidase (HRP) Detection System (Biocare Medical, Pacheco, CA, USA). Immunohistochemistry slides were scanned with a 3DHITECH PANNORAMIC Midi slide scanner, and images were captured using PANNORAMIC Viewer software (3DHITECH, Budapest, Hungary).

### Data analysis

Data for in vitro experiments are expressed as mean ± SD. Data for in vivo experiments are expressed as mean ± standard error of the mean (SEM). Differences in mean values between groups were analyzed through a nonparametric *t* test. One-way analysis of variance (ANOVA) test, followed by Bonferroni posttest comparison, was employed for multiple comparison analysis. Kaplan–Meier survival curves were analyzed using Mantel–Cox test. A *p* value of < 0.05 indicated significant differences. We conducted the statistical analyses using GraphPad Prism 5.

## Results

### Antitumor effect of DBPR114 on human liver cancer cell lines in vitro

**Fig. 1:**
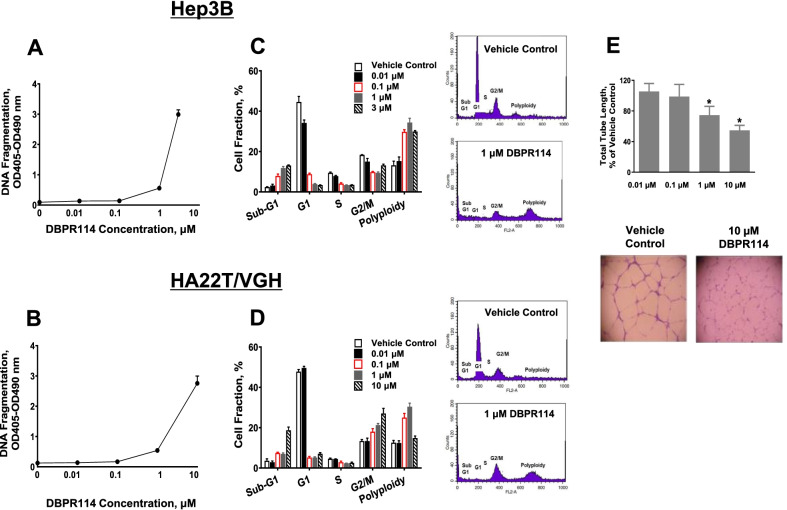
Effect of DBPR114 on apoptosis induction, cell cycle distribution, and HUVEC tube formation. **A** and **B** Apoptosis induction. Hep3B and HA22T/VGH cells were treated with DBPR114 at the indicated concentrations for 48 h in 10% FBS-containing cell culture medium. Apoptotic cell death was measured using DNA fragmentation ELISA. Mean ± SD, n = 3 replicates per concentration. **C** and **D** Cell cycle distribution. Cells were treated with DBPR114 in the presence of 10% FBS-containing cell culture medium for 48 h and then stained with propidium iodide. Cell cycle distribution was assessed using flow cytometry and quantified using FlowJo software. Mean ± SD, n = 3 replicates per concentration. Representative flow cytometry plots are presented for vehicle control and 1 μM DBPR114 from three replicates. **E** Tube formation of HUVEC. HUVEC were treated with DBPR114 at the indicated concentrations for 18 h. The tube formation was imaged, and the tube length was measured. Mean ± SD, n = 3 replicates per concentration. *p < 0.05 vs. vehicle control measured using unpaired *t* test

**Fig. 2:**
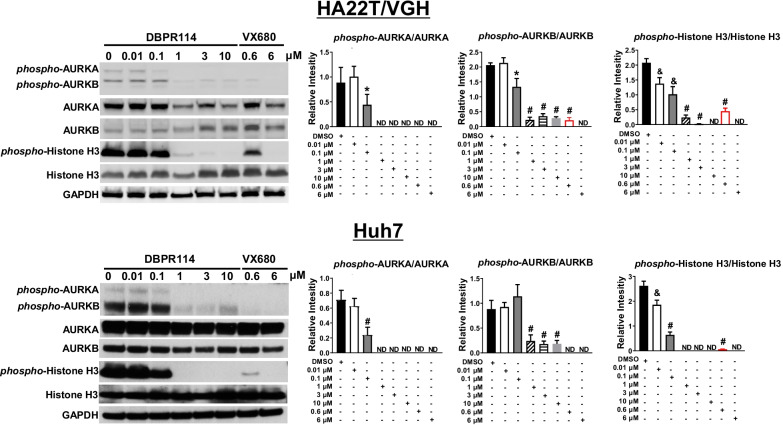
Effect of DBPR114 on phosphorylation of AURKs and histone H3 in human HCC cells. HA22T/VGH and Huh7 cells were pretreated with nocodazole for 16 h prior to DBPR114 treatment lasting 2 h at the indicated concentrations. VX680 was used as the positive control for AURKA and AURKB and histone H3 phosphorylation. At the end of drug treatment, cells were lysed, and the soluble protein was separated using electrophoresis on a sodium dodecyl sulfate and polyacrylamide gel and analyzed through Western blotting. Autoradiographs were scanned for densitometric analysis using Image J software. Quantitation of the protein band was determined through normalization with the internal control GAPDH. Each bar represents the average value of three experiments. ND: not detected. Representative gel images are presented from three independent experiments. *p < 0.05 vs. DMSO-treated cells, ^&^*p* < 0.01 vs. DMSO-treated cells, ^#^*p* < 0.001 vs. DMSO-treated cells, measured using nonparametric *t* test

To determine the utility of DBPR114 in the treatment of HCC, we first evaluated the in vitro growth inhibition of DBPR114 against a panel of liver cancer cell lines on the basis of histopathology/genetic background including tumor grade, tumor subtypes, p53 alteration status, presence of the hepatitis B virus (HBV) and hepatitis C virus (HCV), and similarity in gene expression profiles compared with the human tumor samples [27]. As detailed in Table 1, DBPR114 induced dose-dependent growth inhibition in all the tested cell lines. The antiproliferative efficacy, measured using the IC_50_ values, was approximately 3- to 12-fold more potent than that of sorafenib. DBPR114 also induced apoptotic cell death, which was measured through DNA fragmentation assay (Fig. 1A, B). Cell cycle analysis revealed that DBPR114 treatment resulted in a dose-dependent increase in the sub-G1 population, which is indicative of late-apoptotic or dead cells, and increased polyploidy, indicative of the mitotic checkpoint inhibition of AURKA and AURKB (Fig. 1C, D) [14]. In addition to its effects on tumor cells, DBPR114 also exhibited antiangiogenic activity, as demonstrated by the dose-dependent reduction of endothelial tube formation in the HUVEC (Fig. 1E). Western blotting of the HCC cells indicated that the DBPR114-mediated antitumor effect was associated with the inhibition of phosphorylated AURKA and AURKB and dephosphorylation of the AURK substrate histone H3 at serine 10 (Fig. 2).

**Fig. 3:**
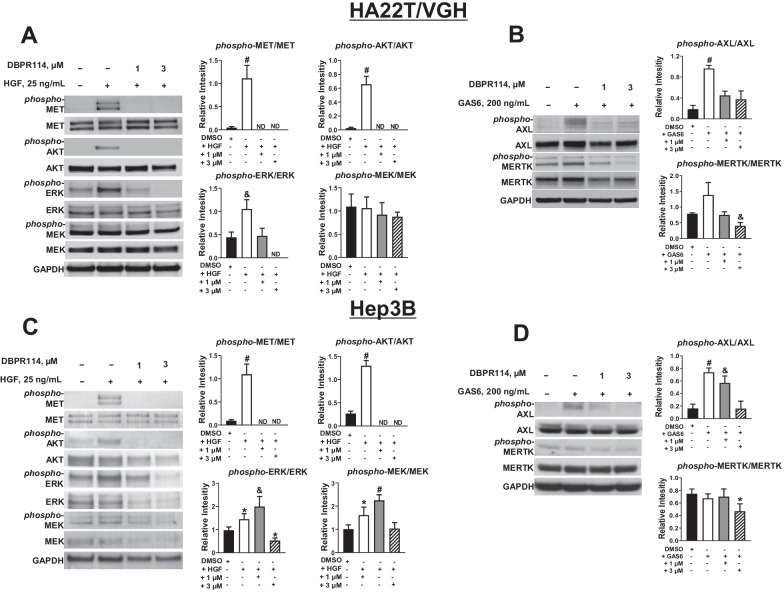
Effect of DBPR114 on receptor tyrosine kinase phosphorylation in human HCC cells. **A** and **C** Modulation of MET receptor tyrosine kinase and downstream effector proteins. HA22T/VGH and Hep3B cells were treated with DBPR114 for 2 h, and 25 ng/mL HGF (ligand for MET) was added 10 min before the end of drug treatment. **B** and **D** Modulation of AXL and MERTK receptor tyrosine kinases. HA22T/VGH and Hep3B cells were treated with DBPR114 in the presence of 200 ng/mL GAS6 (ligand for AXL and MERTK) for 30 min. At the end of drug treatment, cells were lysed, and the soluble protein was separated using electrophoresis on a sodium dodecyl sulfate and polyacrylamide gel and analyzed through Western blotting. Autoradiographs were scanned for densitometric analysis using Image J software. Quantitation of the protein band was determined through normalization with the internal control GAPDH. Each bar represents the average value of three experiments. ND: not detected.  Representative gel images are presented from three independent experiments. *p < 0.05 vs. DMSO-treated cells, ^&^*p* < 0.01 vs. DMSO-treated cells, ^#^*p* < 0.001 vs. DMSO-treated cells, measured using  nonparametric *t* test

Kinase profiling using KINOMEScan has revealed that, in addition to targeting FLT3/AURK signaling pathways, MET and AXL, two kinases that play a critical role in HCC tumor progression, invasion, and metastasis [32, 33], were potently inhibited by DBPR114. AXL belongs to the family of TAM (TYRO3, AXL, and MERTK) receptor tyrosine kinases. Both AXL and MERTK play key roles in tumor cell proliferation, migration, invasion, survival and treatment resistance [34]. To determine whether DBPR114 affected the MET, AXL and MERTK signaling pathway, we first examined the active state of MET, AXL, and MERTK protein expression in HA22T/VGH cells by Western blotting. DBPR114 reduced phosphorylated MET, AXL, and MERTK protein levels (Fig. 3A, B). We also examined protein levels of AKT, ERK, and MEK, three major downstream effectors of MET signaling pathway proteins. Our results showed that the levels of phosphorylated AKT and ERK proteins were also inhibited by DBPR114 in these cells. The level of phosphorylated MEK protein was unchanged by the treatment (Fig. 3A). Similar results were also observed in Hep3B cells (Fig. 3C, D). In the Hep3B cells, the phosphorylated MEK protein level and the total protein levels of AKT, ERK, and MEK were also reduced through DBPR114 treatment. Overall, these results demonstrated that DBPR114 was active against HCC through modulation of the AURK, MET, and AXL/MERTK signaling pathways.

### Antitumor efficacy of DBPR114 in liver cancer xenograft models

**Fig. 4:**
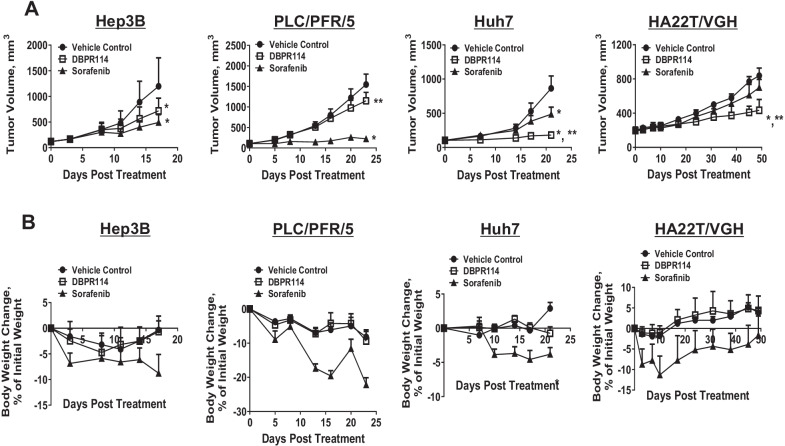
Effect of DBPR114 and sorafenib on growth and body weight changes in human HCC xenograft tumors. **A** Antitumor efficacy and **B** body weight changes from baseline (%). Tumor-bearing animals were randomized; treatment began when the mean tumor volume reached 150 mm^3^. DBPR114 was administered intravenously at 40 mg/kg once a week. Sorafenib was administered at 30 mg/kg once a day, 5 days per week by oral gavage for 3–6 weeks depending on the individual tumor growth rate of each model. Mean ± SEM, n = 7 mice per group for Hep3B and PLC/PRF/5, n = 8 mice per group for Huh7, and n = 12 mice per group for H22T/VGH mice. *p < 0.05 vs. vehicle control, **p < 0.05 vs. sorafenib, measured using  one-way ANOVA and Bonferroni posttest comparison. Error bars in some data points are smaller than the symbols

We next evaluated the anti-tumor effects and responses of DBPR114 and sorafenib using four HCC xenograft tumor models. As presented in Fig. 4A and Table 2, poorly differentiated HA22T/VGH tumors (mutant-type p53 (p53^MT^) and hepatitis B-positive (HBV^+^)) were sensitive to DBPR114 but resistant to sorafenib. Both moderately differentiated Huh7 (p53^MT^ and hepatitis B-negative (HBV^−^))and well differentiated Hep3B xenograft tumors(p53 deletion (p53^null^) and hepatitis B-positive (HBV^+^)) exhibited strong responses to these agents. Moderately differentiated PLC/PRF/5 tumors (p53^MT^ and HBV^+^) were sensitive to sorafenib but unresponsive to DBPR114. Moreover, the antitumor efficacy of DBPR114 was independent of p53 alteration status and HBV positivity. The intravenous administration of 40 mg/kg DBPR114 once a week was well tolerated, as indicated by the less than 10% body weight loss over the course of study in all tumor models tested (Fig. 4B). However, progressive weight loss was observed in the vehicle-treated PLC/PRF/5 animals. The average body weight loss at the end of study was − 9.0% ± 4.0% (Fig. 4B). Thus, the weight loss noted in the DBPR114-treated group was associated with tumor growth and was independent of drug treatment.

**Table 2:** Antitumor activity of DBPR114 and sorafenib in human HCC xenograft tumors

Cell line	Histopathology	Tumor growth inhibition, (TGI), % of vehicle control group		Body weight change, % of initial weight	
		DBPR114	Sorafenib	DBPR114	Sorafenib
HA22T/VGH	HCC, p53^MT^, poorly differentiated, HBV^+^/HCV^−^	54.8 ± 3.8*, **	27.2 ± 2.3	3.9 ± 1	− 1.6 ± 1.2
Huh7	HCC, p53^MT^, moderately differentiated, HBV^−^/HCV^−^	86.7 ± 3.2*	50.6 ± 10.2*	0.7 ± 0.9	− 3.8 ± 0.9
PLC/PRF/5	HCC, p53^MT^, moderately differentiated, HBV^+^/HCV^−^	25.7 ± 13.3**	85.5 ± 1.8*	− 9.4 ± 2.8	− 22.2 ± 2.1
Hep3B	HCC, p53^null^, well differentiated, HBV^+^/HCV^−^	40 ± 6.9*	58.8 ± 4.2*	− 0.7 ± 1.0	− 8.8 ± 1.2

HCC xenograft tumors were treated with DBPR114 (40 mg/kg) once a week intravenously or sorafenib (30 mg/kg) once a day, 5 days per week orally for 3–6 weeks. Mean ± SEM, n = 7–12 mice per group

*p < 0.05 vs. vehicle control, **p < 0.05 vs. sorafenib, measured using one-way ANOVA and Bonferroni posttest comparison

**Fig. 5:**
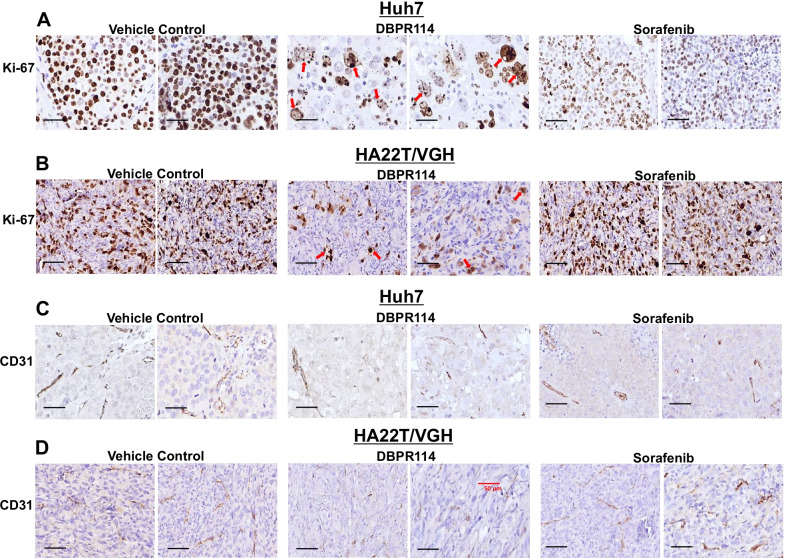
Histologic analysis of DBPR114 and sorafenib treatment in human HCC xenograft tumors. **A** and **B** Effect on cell proliferation (Ki-67 staining) and **C** and **D** effect on vascular endothelial cells (CD31 staining) in Huh7 and HA22T/VGH xenograft tumors. Tumor tissues were formalin-fixed and paraffin-embedded, with the paraffin sections used for immunohistochemical staining. Digital scans were performed with a 3DHITECH PANNORAMIC Midi slide scanner, and images were captured with PANNORAMIC Viewer software. Representative images were extracted from two separate animals in each group at × 40 magnification. Red arrows indicate apoptotic cell death and multinucleated cells. Bar: 50 µm

Histologic analysis in DBPR114-sensitive Huh7 and HA22T/VGH tumors revealed that DBPR114 treatment reduced the frequency of cell proliferation (measured using the proliferation marker Ki-67, Fig. 5A, B) and microvessel density (measured using the endothelial cell marker CD31, Fig. 5C, D). Notably, DBPR114-treated tumors exhibited mitotic arrest, apoptotic cell death, and multinucleated cell formation, as indicated by enlarged cytoplasmic content with enrichment for giant multinucleated cells characteristic of mitotic catastrophe (Fig. 5A, B red arrows). In the Huh7 tumor model, sorafenib treatment reduced cell proliferation and microvessel density. The cell size was reduced, but cellular morphology was similar to that of the control group (Fig. 5A, C). In the HA22T/VGH tumor model, sorafenib had no effect on cell proliferation and microvessel density (Fig. 5B, D). No marked morphological difference was noted between the sorafenib and untreated control group, verifying the lack of antitumor activity in this model. The PLC/PRF/5 xenograft tumors exhibited greater sensitivity to sorafenib than to DBPR114, despite sorafenib showed a threefold higher IC_50_ value than that of DBPR114 in vitro. We noted that PLC/PRF/5 xenograft tumors were highly vascularized compared to Huh7 and HA22T/VGH, as measured by endothelial cell marker CD31, which could make these tumors more sensitive to sorafenib-mediated anti-angiogenic effects (Additional file 1: Fig. S2, lower panel). Sorafenib treatment reduced cell proliferation frequency without altering cell morphology in these tumors (Additional file 1: Fig. S2, upper panel). On the other hand, the DBPR114 treatment partially reduced microvessel density compared with the vehicle-treated group. Tumors treated with DBPR114 exhibited mononucleated and multinucleated giant cells and enlarged empty space in morphology. However, cells that were not affected by DBPR114-mediated mitotic arrest continued to proliferate (as measured by cell proliferation marker Ki-67, Additional file 1: Fig. S2, upper panel).

### Pharmacodynamic biomarker analysis of DBPR114 treatment response

**Fig. 6:**
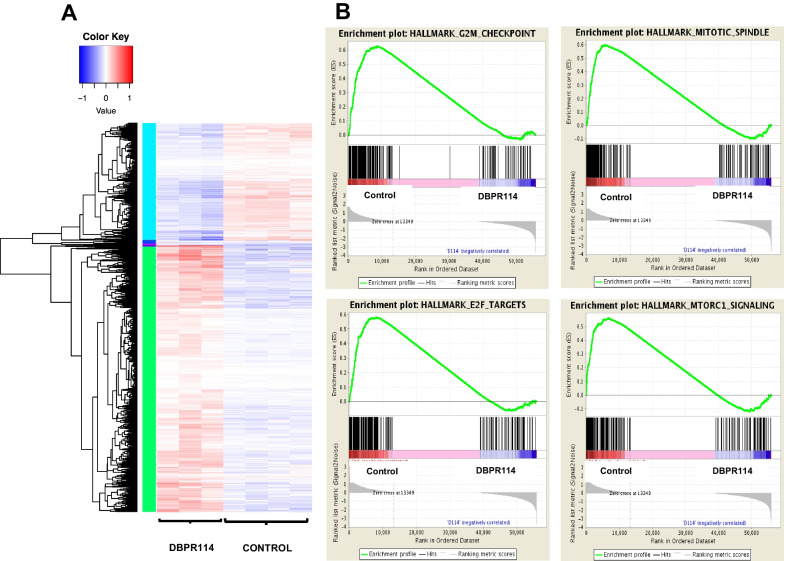
Effect of DBPR114 on gene expression profiles in Huh7 xenograft tumors. **A** Heatmap displaying the differentially expressed genes in DBPR114-treated tumors. Huh7 tumors from the control and treated groups (Fig. 4) were harvested at the end of study. Tumor tissue RNA was isolated and subjected to RNA sequencing analysis using human-specific genome read data. The significantly upregulated and downregulated genes between the treatment and vehicle control groups were selected if they had an FDR < 0.05 and absolute fold change ≥ 2. n = 4 per group for vehicle control, and n = 3 per group for DBPR114. Red: increased; blue: decreased; white: unchanged. **B** GSEA (FDR < 0.05) revealed enrichment of the gene sets of the G2/M checkpoint, mitotic spindle assembly, E2F targets, and mTORC complex 1 signaling following DBPR114 treatment

To further understand the pathways and gene sets regulated in relation to DBPR114-sensitive tumors and identify potential biomarkers associated with DBPR114 treatment response, we performed RNA sequencing analysis on the vehicle control and treated Huh7 tumors harvested at the end of study, as depicted in Fig. 4. A total of 1550 differentially expressed genes (> twofold, *p* < 0.05) were identified between the DBPR114 and vehicle control groups (489 and 1061 downregulated and upregulated genes, respectively; Fig. 6A and Additional file 2: Table S1). GSEA revealed that DBPR114 DBPR downregulated gene sets related to the G2/M checkpoint, mitotic spindle assembly, E2F targets, and mTOR complex 1 signaling reported in the MSigDB (Fig. 6B, Table 3, and Additional file 2: Tables S2A–S2D). Notably, several genes modulated through DBPR114 treatment and identified through GSEA were involved in cell cycle progression and mitotic spindle assembly, namely *BUB1*, *CCNB2*, *CDKN1B*, *CENPE*, *NEK2*, *MCM2*, *MCM4*, *PLK1*, and *PLK4*. These molecular findings together with the observed cellular and histological changes verified the mechanism of DBPR114-mediated drug action.

**Table 3:** Human gene sets significantly downregulated by DBPR114

Gene set	Size	*p* value	FDR	Description	Reference
HALLMARK_G2M_CHEKPOINT	190	0.000	0.000	Genes involved in the G2/M checkpoint that progress through the cell division cycle	MSigDB v5.0
HALLMARK_MITOTIC_SPINDLE	197	0.001	0.001	Genes essential for mitotic spindle assembly	MSigDB v5.0
HALLMARK_E2F_TARGETS	195	0.003	0.002	Genes encoding cell cycle–related targets of E2F transcription factors	MSigDB v5.0
HALLMARK_MTORC_SIGNALING	195	0.001	0.005	Genes upregulated through activation of mTOR complex 1	MSigDB v5.0

Gene set enrichment analysis was performed on Huh7 xenograft tumors (FDR < 0.05). The Huh7 tumors from the vehicle control and treated groups were harvested at the end of studies (Fig. 4). The tumor RNA was isolated and analyzed for human gene expressions through mRNA sequencing

### Effect of DBPR114 on treatment response in sorafenib-refractory and sorafenib-acquired resistant liver cancer tumor models

**Fig. 7:**
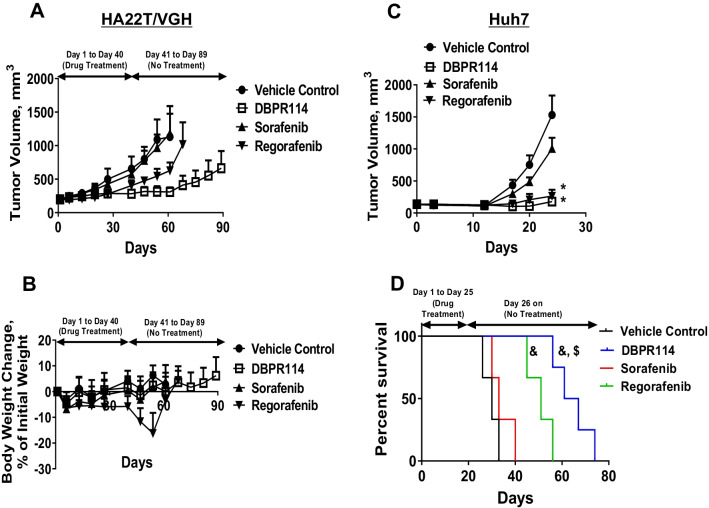
Effect of DBPR114 and regorafenib on sorafenib-refractory and sorafenib-acquired resistant human HCC xenograft tumors. **A** and **B** Tumor growth curves and body weight changes from baseline (%) for the control and treated HA22T/VGH xenograft tumors. **C** Tumor growth curves for the control and treated sorafenib-acquired resistant Huh7 xenograft tumors. **D** Survival in the control and treated sorafenib-acquired resistant Huh7 xenograft tumor groups. DBPR114 (40 mg/kg) was administered once a week intravenously for 6 weeks for the HA22T/VGH xenograft tumors and 3 weeks for the sorafenib-acquired resistant Huh7 xenograft tumors. Sorafenib and regorafenib were administered at 30 mg/kg once a day, 5 days per week by oral gavage for 40 days for the HA22T/VGH xenograft tumors and 25 days for the Huh7 xenograft tumors. Mean ± SEM, n = 8 mice per group for both xenograft tumors. *p < 0.05 vs. vehicle control measured using one-way ANOVA and Bonferroni posttest comparison.  ^&^*p* < 0.01 vs. control, ^$^*p* < 0.05 vs. regorafenib, measured using Mantel–Cox test

On the basis of the in vivo antitumor efficacy evaluation of DBPR114 (Fig. 4A), the HA22T/VGH tumor type was identified as intrinsically resistant to sorafenib. To evaluate the utility of DBPR114 as a second-line treatment for patients with HCC who are refractory to sorafenib, a separate experiment was conducted. The HA22T/VGH tumor-bearing mice were randomized into four groups in which the average tumor volume was 200 mm^3^ and were treated with the vehicle control, sorafenib, DBPR114, or regorafenib for 6 weeks. Sorafenib had no effect on HA22T/VGH tumor growth during the treatment course, validating our previous results (Fig. 7A and Additional file 3: Table S3). Regorafenib was efficacious against HA22T/VGH tumors, having reduced tumor volume by 38% on day 40 (*p* < 0.05 vs. vehicle control). However, tumor growth resumed and progressed after treatment was discontinued. DBPR114 treatment reduced the HA22T/VGH tumor volume by 57% compared with the vehicle-treated animals and by an additional 20% compared with the regorafenib-treated animals (*p* < 0.01 vs. vehicle control and *p* < 0.05 vs. regorafenib, Fig. 7A and Additional file 3: Table S3). Notably, tumor growth continued to be suppressed by DBPR114 for an additional 3 weeks after treatment discontinuation, after which tumor growth resumed. In contrast to DBPR114 and sorafenib, with which no significant body weight loss was induced, regorafenib induced weight loss of approximately 6% during the treatment course (Fig. 7B and Additional file 3: Table S3).

The Huh7 tumor type was sensitive to both DBPR114 and sorafenib, exhibiting a TGI greater than 50% compared with the control under both agents (Fig. 4 and Table 2). To assess the antitumor activity of DBPR114 in tumors that developed sorafenib-acquired resistance, we developed a sorafenib-acquired resistant Huh7 xenograft tumor model through repeated sorafenib treatments in vivo (Additional file 1: Fig. S1). Thereafter, sorafenib-acquired resistant Huh7 tumors were harvested and implanted into recipient mice. These animals were randomized when the average tumor volume reached approximately 100 mm^3^ and were treated with the vehicle control, sorafenib, DBPR114, or regorafenib for 25 days. As expected, sorafenib treatment was ineffective against sorafenib-acquired resistant Huh7 tumors. The average tumor volume on day 25 was 1531 ± 302 mm^3^ and 1,004 ± 169 mm^3^ for the vehicel control and sorafenib-treated groups, respectively (Fig. 7C and Additional file 3: Table S4). DBPR114 and regorafenib were equally efficacious against sorafenib-resistant Huh7 tumors, reducing tumor volumes by 82.7% ± 2.4% and 88% ± 1%, respectively, compared with the vehicle control at the end of 3-week treatment (*p* < 0.05 vs. vehicle control for both treatment groups; Fig. 7C and Additional file 3: Table S4). Treatment was discontinued at the end of drug treatment, and the animals were monitored for tumor growth and body weight changes, as detailed in Additional file 1: Fig. S3. As illustrated in Fig. 7D, the median survival time for the vehicle-treated and sorafenib-treated group was 30 and 33.5 days, respectively; regorafenib treatment prolonged the median survival to 51.5 days compared with the vehicle-treated group (*p* < 0.01). Notably, DBPR114 treatment significantly delayed tumor recurrence and improved posttreatment survival (median survival time = 65.5 days) compared with the vehicle control and regorafenib groups (*p* < 0.01 and *p* < 0.05, respectively; Fig. 7D). These findings demonstrated that DBPR114 was efficacious as a second-line therapy against both sorafenib-refractory and sorafenib-acquired resistant HCC tumors. In addition, DBPR114 delayed tumor regrowth and prolonged posttreatment survival compared with the current second-line agent regorafenib.

## Discussion

HCC is the most common liver cancer and one of the deadliest cancers worldwide [1]. Most patients with HCC are diagnosed at the advanced stage when therapeutic options are limited. Furthermore, this disease often recurs following localized treatment [3]. The lack of potent and effective therapies, particularly at the advanced stage, is a primary reason for the poor prognosis of patients with HCC. Current FDA-approved first- and second-line agents target VEGFR (tumor angiogenesis), PDGFR (metastasis), and RAS/RAF/MAP/ERK (oncogenesis) signaling pathways. Because these agents have similar mechanisms of action and overlapping target kinase profiles, only a subset of patients can be benefit from these therapies, which leaves few options for patients who are insensitive to these agents or who develop acquired resistance to these targeted therapies.

The AURK family of serine/threonine kinases involve various mitotic activities during cell division and maintain the integrity of the genome [13]. AURKs are frequently overexpressed in certain tumors, including HCC tumors (see reviews in [14]). Together with the association of AURKs with genetic instability and aneuploidy in tumors, the findings of these studies indicated that anticancer agents targeting mitosis during cell division through the inhibition of AURKs have remarkable potential for cancer treatment [35]. Furthermore, because AURKs are frequently overexpressed in various cancer types, a wide range of cancers could respond therapeutically to AURK inhibitors. Both AURKA and AURKB are highly expressed in HCC, and overexpression is associated with tumor aggressiveness, an unfavorable prognosis, and poorer outcomes [15–17]. These findings revealed the potential of AURKs as targets for the treatment of HCC. We previously have reported the development of a FLT3/AURK dual multikinase inhibitor that was potent against FLT3 overexpression in AML and multiple solid tumor types [26]. To further explore the utility of DBPR114 as a multikinase inhibitor in the treatment of liver cancer in the present study, we used a panel of human liver cancer cell lines with similar histopathology/genetic backgrounds to human HCC tumors to evaluate the antitumor efficacy of DBPR114 in human HCC tumors. Potential pharmacodynamic biomarkers associated with treatment response were also investigated. We demonstrated that DBPR114 was active against both HBV^+^ and HBV^−^ HCC tumors in vitro and in vivo. The antitumor activity of DBPR114 was independent of the status of the tumor suppressor p53 and tumor grade. Because p53 is frequently altered in patients with HCC, and more than 50% of these patients have the HBV virus [3], these results suggest that the majority of patients could potentially benefit from DBPR114 treatment.

To understand the mechanisms underlying the DBPR114-mediated antitumor effect, we first evaluated the effect of DBPR114 on cell death and cell cycle progression in vitro. In both the well-differentiated Hep3B cell line and poorly differentiated HA22T/VGH cell line, DBPR114-mediated growth inhibition was associated with dose-dependent apoptosis induction, cell cycle arrest, and polyploidy formation. Further analysis of the HCC cells revealed that DBPR114 reduced phosphorylated AURKA and AURKB proteins and the AURK substrate histone H3 at serine 10. The inhibition of both AURKA and AURKB induced apoptosis through distinct mechanisms [36]. AURKA plays a pivotal role in centrosome maturation, bipolar spindle assembly, and chromosome separation, and its inhibition causes transient spindle checkpoint-dependent mitotic arrest. AURKA-inhibited cells can exit from mitosis, leading to an accumulation of apoptotic cells at the sub-G1 phase. AURKB is involved in chromosome condensation and regulates the spindle checkpoint and cytokinesis. Its inhibition interferes with normal chromosome alignment during mitosis and overrides the mitotic spindle checkpoint, which results in polyploidy, cytokinesis failure, and endoreduplication followed by cell death. Flow cytometry analysis indicated that DBPR114 treatment increased the sub-G1 cell population and polyploid cells, and histologic analysis of DBPR114-treated HCC xenograft tumors revealed an increased frequency of mitotic cell arrest, apoptotic cell death, and multinucleated cell formation. Furthermore, GSEA of DBPR114-treated Huh7 xenograft tumors revealed that several genes were involved in cell cycle progression and mitotic spindle assembly. Collectively, these in vitro and in vivo findings are consistent with the consequences of the inhibition of AURKA and AURKB activities. Our data from the HCC cell lines and xenograft tumors further verified the mechanism of action of DBPR114 reported previously in AML and colon cancer cell lines, in which DBPR114 modulated FLT3 and AURKA/B inside the cells and induced the accumulation of multinucleated cells [26].

As mentioned previously, the initial response to sorafenib was modest, with sorafenib resistance developing over time. Regorafenib is currently the second-line therapy for patients who have relapsed after sorafenib treatment based on the positive results obtained from the phase 3 Study of Regorafenib After Sorafenib in Patients with Hepatocellular Carcinoma (NCT01774344). Because of its similar mode of action to sorafenib, regorafenib is not suitable for patients who are intolerant to sorafenib (see reviews in [5]). We assessed the effect of DBPR114 on sorafenib-resistant HCC tumors and compared its antitumor efficacy with that of the current second-line agent regorafenib. Our data indicated that DBPR114 was efficacious against sorafenib-intrinsic and -acquired resistant HCC tumors, more significantly delaying post treatment tumor regrowth and prolonging survival compared with regorafenib. Collectively, our findings indicated that agents that modulate mitotic arrest may be beneficial for sorafenib-refractory and sorafenib-relapsed patients through the delay or prevention of tumor recurrence and treatment resistance.

In this study, we demonstrated that, in addition to targeting AURK signaling, DBPR114 was also active against the MET and AXL signaling pathways in HCC cell lines. Both the MET and AXL signaling pathway play crucial roles in HCC tumor progression, invasion, and metastasis [32, 33], with their overexpression acting as predictors of poor prognosis in patients with HCC [37, 38]. Furthermore, both MET and AXL are involved in resistance to antiangiogenic agents in renal cell carcinoma [39]; overexpression of these kinases was observed in HCC tumors that developed acquired resistance to sorafenib [40, 41]. In the aforementioned studies, sorafenib-resistant cell lines were developed through the exposure of HCC tumor cells to increasing concentrations of sorafenib in vitro. The resistant cell clones were then selected for the study of the mechanisms of sorafenib resistance. We also produced sorafenib-acquired resistant HCC tumors through repeated treatment with therapeutic doses of sorafenib in tumor-bearing mice in vivo until the tumors became refractory to sorafenib. Because we did not characterize the MET- or AXL-induced development of sorafenib-acquired resistant tumors, our model requires further examination.

FLT3, a type III receptor tyrosine kinase, is essential in the regulation of normal hematopoietic cell function and is frequently altered in AML [42]. In addition to its role in hematopoietic lineage, FLT3 acts as a biomarker for hepatic oval cells that have differentiated into hepatocytes and bile duct lineages in rodents [43]. The role of FLT3 in hematopoiesis and liver development indicates that FLT3 signaling may have a crucial function in liver regeneration. Aydin et al. [44] have reported that FLT3 was active and participatory in the proliferation response during progenitor-dependent liver regeneration in rats. The role of FLT3 in HCC tumorigenesis, proliferation, and invasion was demonstrated through the stable knockdown of *FLT3* gene in the FLT3-expressing HCC cell line and the pharmacological inhibition of FLT3 kinase [45]. According to the Cancer Genome Atlas dataset [46], *FLT3* gene expression level and copy number gain is associated with the low OS of patients with HCC, implying that FLT3 may play a key role in HCC progression. However, Sun et al. [47] have reported that FLT3 gene and protein expression was significantly decreased in specimens from patients with HCC compared with that in adjacent normal liver tissue. This reduced expression of FLT3 in HCC was attributed to the frequent FLT3 copy number losses. Notably, although the prognosis of patients with HCC and high FLT3 levels or copy number gains was poor, high FLT3 levels were significantly correlated with the improved OS of patients with HCC undergoing sorafenib treatment [47]. In our study, the FLT3 protein expression level in the tested HCC cells was not detected through Western blotting. In addition, the cellular, histological, and molecular changes in drug-treated tumors indicated that the DBPR114-mediated antitumor effect was primarily a result of the inhibition of AURK activity. These data indicated that FLT3 signaling may minimally affect DBPR114-mediated growth inhibition in the HCC cell lines. Further investigation into the antitumor effect of DBPR114 in FLT3-expressing HCC cells is warranted.

Biomarkers are critical clinical tools in the monitoring of treatment effects, indicating whether the test substance is having the desired biological effect on target tissues. The incorporation of biomarkers and surrogate endpoints into oncological drug development is essential for guiding comprehensive drug development and regulatory decisions [48]. Our research on HCC cell lines demonstrated that DBPR114 inhibited HCC tumor cell growth, induced apoptotic cell death, and modulated MET receptor tyrosine kinase activities. The unique morphologic features of apoptotic cell death and multinucleated cell formation induced by DBPR114 as well as DBPR114-modulated mitotic checkpoint- and spindle assembly-associated gene signatures were consistent with the activity of AURK inhibition. DBPR114-mediated cellular and molecular changes could potentially be used as pharmacodynamic biomarkers to verify the mechanisms of drug action, ensure adequate target engagement, and serve as intermittent endpoints for the early indication of treatment efficacy. In this study, HCC xenograft tumors exhibited differential sensitivity to the DBPR114-mediated antitumor effect. Notably, the PLC/PRF/5 xenograft tumors were insensitive to DBPR114 (TGI = 25.7%) but highly sensitive to sorafenib (TGI = 85.5%) in vivo, and DBPR114 was three times more potent than sorafenib in inhibiting PLC/PRF/5 tumor cell growth in vitro. This discrepancy between the in vitro and in vivo findings may be partially attributable to the highly vascularized nature of PLC/PRF/5 tumors (Additional file 1: Fig. S2), which may be more prone to sorafenib-mediated antiangiogenic effects. These findings indicated that HCC tumors with high vasculatures may be more suitable for VEGFR-targeted therapy. Further studies are required to determine whether the degree of vascularization and AURKs expression levels can be used as potential biomarkers for patient selection for DBPR114 treatment.

The TAM family of receptor tyrosine kinases constitutes a unique set of antitumor targets. Our in vitro studies demonstrated that DBPR114 inhibited TAM ligand GAS6-mediated AXL and MERTK phosphorylation (Fig. 3). In addition to their function as direct tumor drivers, TAM receptor tyrosine kinases have also been recognized as potential negative immune regulators that suppress host tumor immune responses through multiple mechanisms including the efficient clearance of intracellular antigens, polarization of macrophages toward the M2 phenotype through apoptotic cell debris, dampening of the toll-like receptor (TLR) inflammatory response through AXL signaling, inhibition of the NK cell-mediated antimetastatic effect, and inhibition of activated T cells, TLR signaling, and proinflammatory cytokines (see review in [34]). Many small-molecule kinase inhibitors that are ATP-competitive compounds for receptor tyrosine kinase inhibition also exhibit various degrees of blocking activity against TAM receptor tyrosine kinases, including cabozantinib (VEGFR1-3, MET, AXL, KIT, and RET), sitravatinib (VEGFR2, PDGFRα, AXL, MERTK, RET, MET, tropomyosin receptor kinase A (TRKA), and Discoidin domain receptor 2 (DDR2)), and glesatinib (VEGFR2, MET, recepteur d'Origine nantais (RON), and AXL) [49]. Future research can focus on investigating the role of DBPR114 in the modulation of the tumor immune microenvironment.

## Conclusions

In summary, our preclinical studies indicated that targeting AURK signaling could be a new effective molecular-targeted agent in the treatment of patients with HCC. Furthermore, DBPR114-mediated multi-targeted kinase inhibition may translate into better efficacy, and ultimately, OS for tough to treat solid tumors such as advanced HCC.

## Supplementary Information

**Additional file 1: Figure S1.** Development of sorafenib-acquired resistant Huh7 tumors. Huh7 tumor-bearing animals were treated with 30 mg/kg sorafenib once a day, 5 days per week by oral route for 4 weeks. Tumor-bearing animals with a volume increase of ≥ 30% during treatment were removed at the end of study. The remaining animals with sorafenib treatment-sensitive tumors, that is, tumors with a volume increase of < 30% or regressive tumors, were harvested and reimplanted into recipient mice. These animals were randomized when the average tumor size reached 100 mm^3^ and received sorafenib treatment for 4 weeks. This process was repeated twice when tumors developed acquired resistance to sorafenib. Acquired resistance development is defined as responsive to sorafenib for at least 2 weeks but then exhibited a > 30% increase in tumor volume within 5 days. **Figure S2.** Histologic analysis of DBPR114 and sorafenib treatment in PLC/PRF/5 xenograft tumors. Tumor tissues were formalin-fixed and paraffin-embedded, with the paraffin sections used for immunohistochemical staining. Cell proliferation was measured using the proliferation marker Ki-67 (upper panel), and microvessel density was measured using the endothelial cell marker CD31 (bottom panel). Digital scans were performed with a 3DHITECH PANNORAMIC Midi slide scanner, and images were captured with PANNORAMIC Viewer software. Representative images were extracted from two separate animals in each group at × 40 magnification. Red arrows indicate mononucleated and multinucleated giant cells. Bar: 50 µm. **Figure S3.** Individual animal tumor growth curve for the control and treated sorafenib-acquired resistant Huh7 xenograft tumors. The mice bearing sorafenib-acquired resistant Huh7 tumors were randomized and treated with the vehicle control, sorafenib, DBPR114, or regorafenib when the average tumor size reached 100 mm^3^. DBPR114 (40 mg/kg) was administered once a week intravenously for 3 weeks. Sorafenib and regorafenib were administered at 30 mg/kg once a day, 5 days per week by oral gavage for 25 days. The treatment was then discontinued, and the animals were monitored for tumor growth (A) and body weight change (B). n = 8 mice per group.

**Additional file 2: Table S1.** List of differentially expressed genes by DBPR114-treated Huh7 tumors. Tumor tissue RNA was isolated and subjected to RNA sequencing analysis using human-specific genome read data. The significantly upregulated and downregulated genes between the treatment and vehicle control groups were selected if they had an FDR < 0.05 and absolute fold change ≥ 2. n = 4 per group for vehicle control, and n = 3 per group for DBPR114. **Table S2. **List of down-regulated genes associated with (A) E2F targets gene set, (B) G2/M checkpoint gene set, (C) mitotic spindle gene set, and (D) MTORC complex 1 signaling gene set by DBPR114-treated Huh7 tumors. The Huh7 tumors from the vehicle control and treated groups were harvested at the end of studies (Figure 4). The tumor RNA was isolated and analyzed for human gene expressions through mRNA sequencing. Gene set enrichment analysis was performed on Huh7 xenograft tumors (FDR < 0.05).

**Additional file 3: Table S3.** Anti-tumor activity of sorafenib, regorafenib and DBPR114 in sorafenib-refractory HA22T/VGH xenograft tumors on day 40. HA22T/VGH tumor-bearing mice were treated with 40 mg/kg DBPR114 once a week intravenously for 6 weeks or sorafenib and regorafenib at 30 mg/kg once a day, 5 days per week orally for 40 days. Mean ± SEM, n = 8 mice per group. *p < 0.05 vs. vehicle control, **p < 0.05 vs. regorafenib, measured using  one-way ANOVA and Bonferroni posttest comparison. **Table S4.** Anti-tumor activity of sorafenib, regorafenib and DBPR114 in sorafenib-acquired resistant Huh7 xenograft tumors on day 25. Sorafenib-acquired resistant Huh7 tumor-bearing mice were treated with 40 mg/kg DBPR114 once a week intravenously for 3 weeks or sorafenib and regorafenib at 30 mg/kg once a day, 5 days per week orally for 25 days. Mean ± SEM, n = 8 mice per group. *p < 0.05 vs. vehicle control measured using one-way ANOVA and Bonferroni posttest comparison.

## Data Availability

The data presented in the study are included in the article and supplementary material.

## Abbreviations

AKT: Protein kinase B
AML: Acute myeloid leukemia
ANOVA: Analysis of variance
AURKA: Aurora kinase A
AURKB: Aurora kinase B
AURKC: Aurora kinase C
AURKs: Aurora kinases
D5W: 5% Dextrose in water
DDR2: Discoidin domain receptor 2
DMEM: Dulbecco's Modified Eagle Medium
DMSO: Dimethyl sulfoxide
EGF: Epidermal growth factor
EGFR: Epidermal growth factor receptor
ELISA: Enzyme-linked immunosorbent assay
ERK: Extracellular signal-regulated kinase
FBS: Fetal bovine serum
FDA: Food and drug administration
FDR: False discovery rate
FGFR: Fibroblast growth factor receptor
FLT3: FMS-like tyrosine kinase 3
GAPDH: Glyceraldehyde 3-phosphate dehydrogenase
GSEA: Gene set enrichment analysis
HBV: Hepatitis B virus
HBV^+^: Hepatitis B-positive virus
HBV^−^: Hepatitis B-negative virus
HCC: Hepatocellular carcinoma
HCV: Hepatitis C virus
HCV^−^: Hepatitis C-negative virus
HGF: Hepatocyte growth factor
HRP: Horseradish peroxidase
HUVEC: Human umbilical vein endothelial cells
IGF: Insulin-like growth factor
IGFR: Insulin-like growth factor receptor
KIT: KIT proto-oncogene receptor tyrosine kinase
MEK: Mitogen-activated protein kinase kinase
MERTK: MER proto-oncogene tyrosine kinase
MET: Mesenchymal–epithelial transition factor
mTOR: Mammalian target of rapamycin
NF-κB: Nuclear factor-kappa B
NOD/SCID: Non-obese diabetic, severe combined immunodeficiency
ND: Not detected
OS: Overall survival
p53^null^: p53 Deletion
p53^MT^: Mutant-type p53
PBS: Phosphate buffered saline
PDGF: Platelet-derived growth factor
PDGFR: Platelet-derived growth factor receptor
PDGFRα: Platelet-derived growth factor receptor alpha
PDGFRβ: Platelet-derived growth factor receptor beta
PFS: Progression-free survival
PI3K: Phosphatidylinositol-3-kinase
PTEN: Phosphatase and tensin homologue deleted on chromosome ten
RET: Rearranged during transfection
RON: Recepteur d'Origine nantais
SD: Standard deviation
SEM: Standard error of the mean
TAM: TYRO3, AXL, MERTK
TGI: Tumor growth inhibition
TIE-2: Tunica interna endothelial-2
TLR: Toll-like receptor
TRKA: Tropomyosin receptor kinase A
TTP: Time to progression
VEGF: Vascular endothelial growth factor
VEGFR: Vascular endothelial growth factor receptor
