# Supplementary material for: Novel FLT3/AURK multikinase inhibitor is efficacious against sorafenib-refractory and sorafenib-resistant hepatocellular carcinoma
Source: J Biomed Sci. 2022 Jan 21;29:5. doi: 10.1186/s12929-022-00788-0 (PMC8781143; doi:10.1186/s12929-022-00788-0)
Supplement: Supplementary file 1 — Additional file 1: Figure S1. Development of sorafenib-acquired resistant Huh7 tumors. Huh7 tumor-bearing animals were treated with 30 mg/kg sorafenib once a day, 5 days per week by oral route for 4 weeks. Tumor-bearing animals with a volume increase of ≥ 30% during treatment were removed at the end of study. The remaining animals with sorafenib treatment-sensitive tumors, that is, tumors with a volume increase of < 30% or regressive tumors, were harvested and reimplanted into recipient mice. These animals were randomized when the average tumor size reached 100 mm3 and received sorafenib treatment for 4 weeks. This process was repeated twice when tumors developed acquired resistance to sorafenib. Acquired resistance development is defined as responsive to sorafenib for at least 2 weeks but then exhibited a > 30% increase in tumor volume within 5 days. Figure S2. Histologic analysis of DBPR114 and sorafenib treatment in PLC/PRF/5 xenograft tumors. Tumor tissues were formalin-fixed and paraffin-embedded, with the paraffin sections used for immunohistochemical staining. Cell proliferation was measured using the proliferation marker Ki-67 (upper panel), and microvessel density was measured using the endothelial cell marker CD31 (bottom panel). Digital scans were performed with a 3DHITECH PANNORAMIC Midi slide scanner, and images were captured with PANNORAMIC Viewer software. Representative images were extracted from two separate animals in each group at × 40 magnification. Red arrows indicate mononucleated and multinucleated giant cells. Bar: 50 µm. Figure S3. Individual animal tumor growth curve for the control and treated sorafenib-acquired resistant Huh7 xenograft tumors. The mice bearing sorafenib-acquired resistant Huh7 tumors were randomized and treated with the vehicle control, sorafenib, DBPR114, or regorafenib when the average tumor size reached 100 mm3. DBPR114 (40 mg/kg) was administered once a week intravenously for 3 weeks. Sorafenib and regorafenib were ad [file 12929_2022_788_MOESM1_ESM.pptx]

## Slide 1
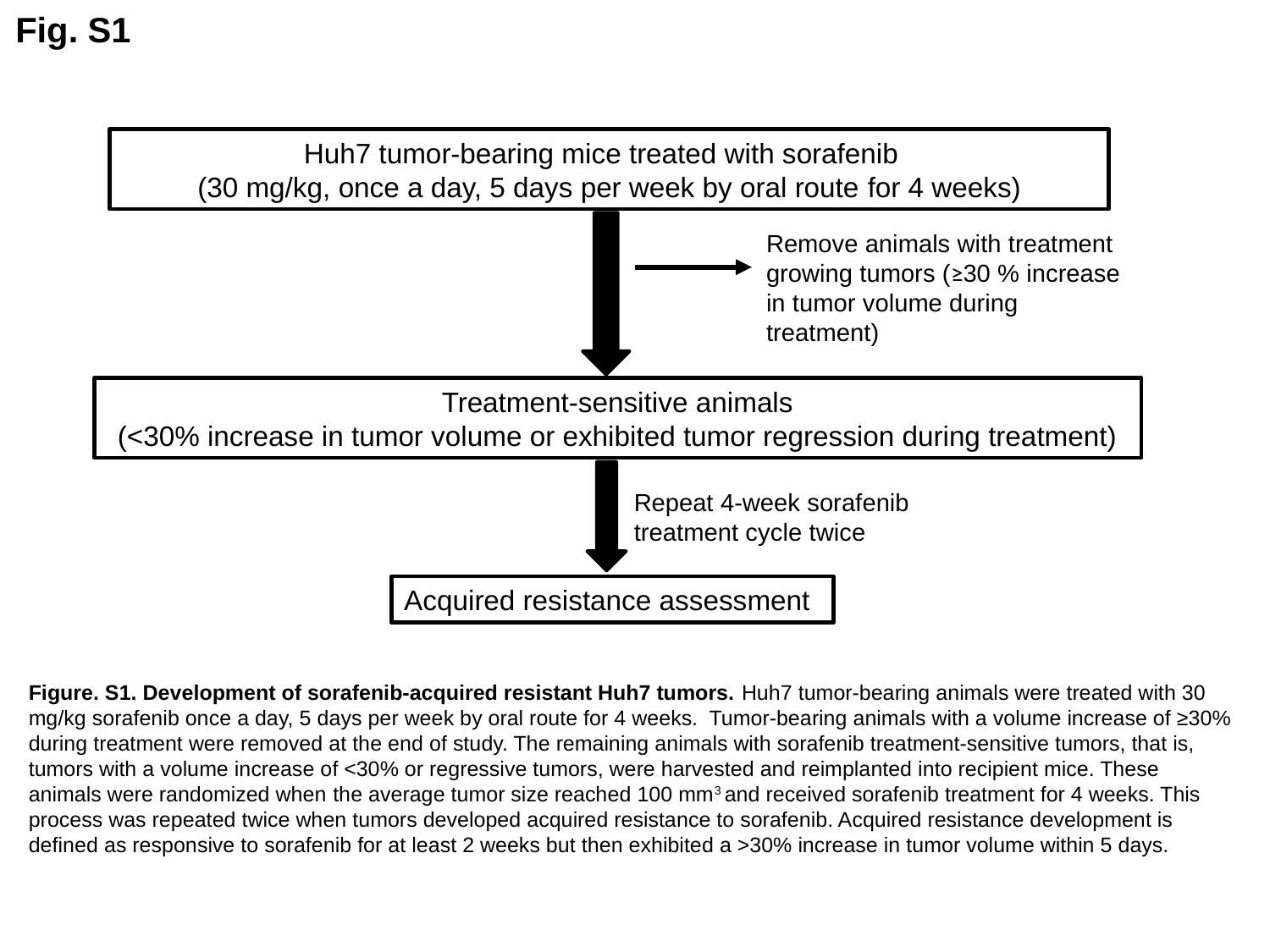

Fig. S1
Huh7 tumor-bearing mice treated with sorafenib
(30 mg/kg, once a day, 5 days per week by oral route for 4 weeks)
Remove animals with treatment growing tumors (≥30 % increase in tumor volume during treatment)
Treatment-sensitive animals
(<30% increase in tumor volume or exhibited tumor regression during treatment)
Repeat 4-week sorafenib treatment cycle twice
Acquired resistance assessment
Figure. S1. Development of sorafenib-acquired resistant Huh7 tumors. Huh7 tumor-bearing animals were treated with 30 mg/kg sorafenib once a day, 5 days per week by oral route for 4 weeks. Tumor-bearing animals with a volume increase of ≥30% during treatment were removed at the end of study. The remaining animals with sorafenib treatment-sensitive tumors, that is, tumors with a volume increase of <30% or regressive tumors, were harvested and reimplanted into recipient mice. These animals were randomized when the average tumor size reached 100 mm3 and received sorafenib treatment for 4 weeks. This process was repeated twice when tumors developed acquired resistance to sorafenib. Acquired resistance development is defined as responsive to sorafenib for at least 2 weeks but then exhibited a >30% increase in tumor volume within 5 days.

## Slide 2
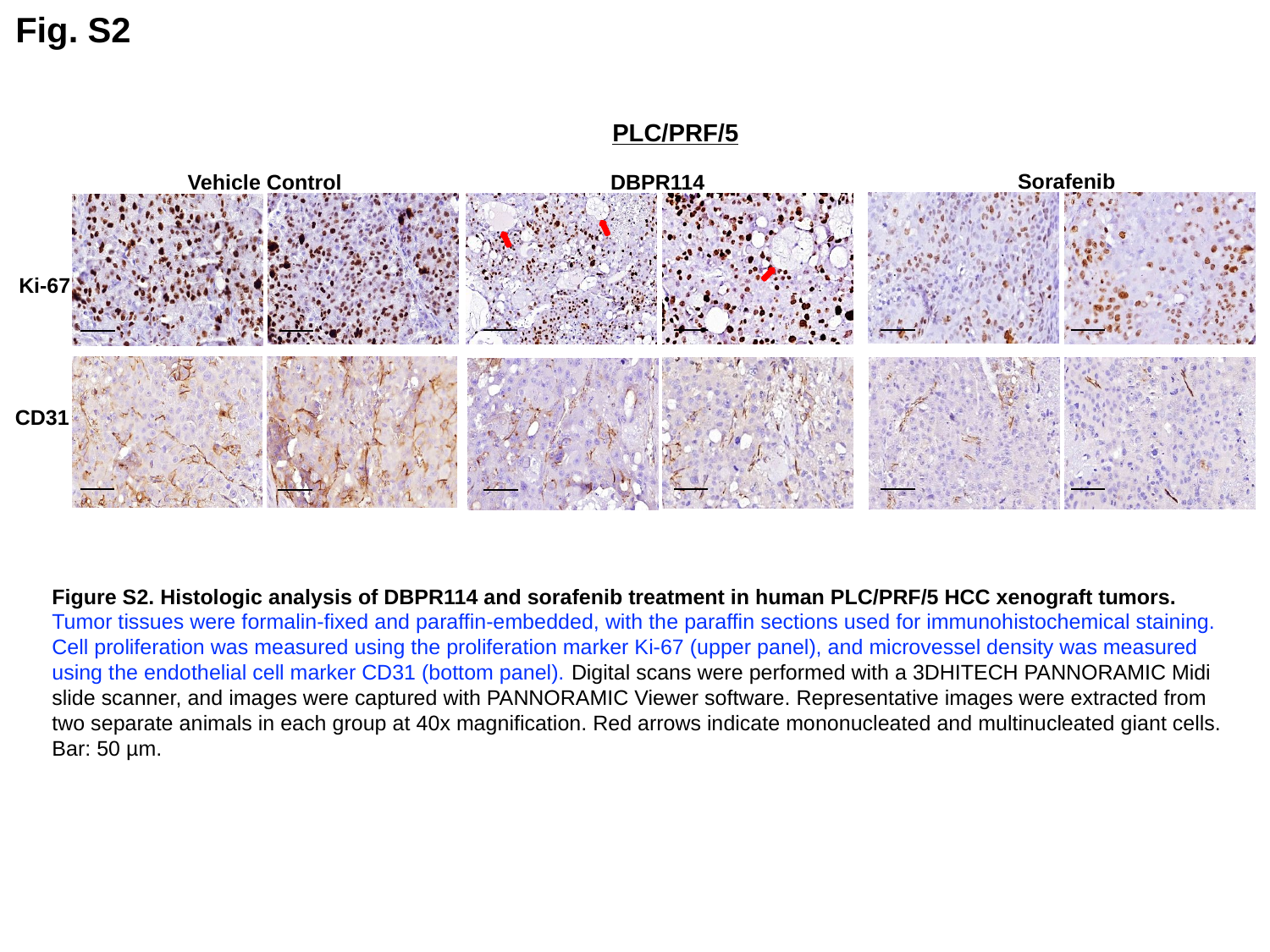

Fig. S2
PLC/PRF/5
Sorafenib
Vehicle Control
DBPR114
Ki-67
CD31
Figure S2. Histologic analysis of DBPR114 and sorafenib treatment in human PLC/PRF/5 HCC xenograft tumors. Tumor tissues were formalin-fixed and paraffin-embedded, with the paraffin sections used for immunohistochemical staining. Cell proliferation was measured using the proliferation marker Ki-67 (upper panel), and microvessel density was measured using the endothelial cell marker CD31 (bottom panel). Digital scans were performed with a 3DHITECH PANNORAMIC Midi slide scanner, and images were captured with PANNORAMIC Viewer software. Representative images were extracted from two separate animals in each group at 40x magnification. Red arrows indicate mononucleated and multinucleated giant cells. Bar: 50 µm.

## Slide 3
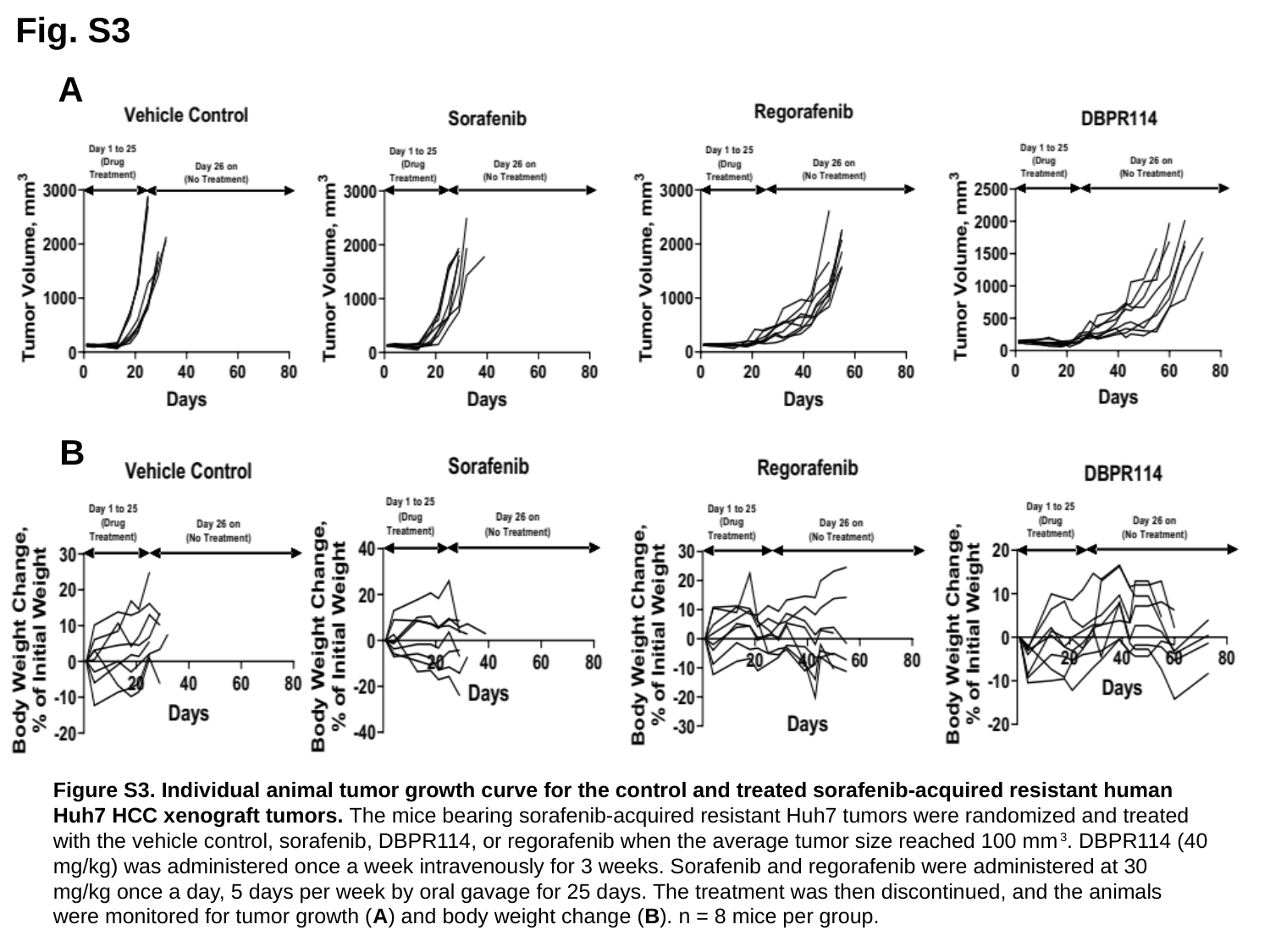

Fig. S3
A
B
Figure S3. Individual animal tumor growth curve for the control and treated sorafenib-acquired resistant human Huh7 HCC xenograft tumors. The mice bearing sorafenib-acquired resistant Huh7 tumors were randomized and treated with the vehicle control, sorafenib, DBPR114, or regorafenib when the average tumor size reached 100 mm3. DBPR114 (40 mg/kg) was administered once a week intravenously for 3 weeks. Sorafenib and regorafenib were administered at 30 mg/kg once a day, 5 days per week by oral gavage for 25 days. The treatment was then discontinued, and the animals were monitored for tumor growth (A) and body weight change (B). n = 8 mice per group.
