# Supplementary material for: Novel FLT3/AURK multikinase inhibitor is efficacious against sorafenib-refractory and sorafenib-resistant hepatocellular carcinoma
Source: J Biomed Sci. 2022 Jan 21;29:5. doi: 10.1186/s12929-022-00788-0 (PMC8781143; doi:10.1186/s12929-022-00788-0)
Supplement: Supplementary file 3 — Additional file 3: Table S3. Anti-tumor activity of sorafenib, regorafenib and DBPR114 in sorafenib-refractory HA22T/VGH xenograft tumors on day 40. HA22T/VGH tumor-bearing mice were treated with 40 mg/kg DBPR114 once a week intravenously for 6 weeks or sorafenib and regorafenib at 30 mg/kg once a day, 5 days per week orally for 40 days. Mean ± SEM, n = 8 mice per group. *p < 0.05 vs. vehicle control, **p < 0.05 vs. regorafenib, measured using one-way ANOVA and Bonferroni posttest comparison. Table S4. Anti-tumor activity of sorafenib, regorafenib and DBPR114 in sorafenib-acquired resistant Huh7 xenograft tumors on day 25. Sorafenib-acquired resistant Huh7 tumor-bearing mice were treated with 40 mg/kg DBPR114 once a week intravenously for 3 weeks or sorafenib and regorafenib at 30 mg/kg once a day, 5 days per week orally for 25 days. Mean ± SEM, n = 8 mice per group. *p < 0.05 vs. vehicle control measured using one-way ANOVA and Bonferroni posttest comparison. [file 12929_2022_788_MOESM3_ESM.docx]

**Table S3. Anti-tumor activity of sorafenib, regorafenib and DBPR114 in sorafenib-refractory human HA22T/VGH HCC xenograft tumors on day 40.** HCC tumors were treated with DBPR114 once a week intravenously for 6 weeks or sorafenib and regorafenib once a day, 5 days per week orally for 40 days. Mean ± SEM, n = 8 mice per group. **p* < 0.05 vs. vehicle control; ***p* < 0.05 vs. regorafenib, measured by one-way ANOVA and Bonferroni posttest comparison.

| **Treatment** | **Tumor volume** | **Tumor growth inhibition (TGI),**  **% of vehicle control group** | **Body weight change, % of initial weight** |
| --- | --- | --- | --- |
| Vehicle control | 653.9 ± 64.7 | - | 4.2 ± 1.4 |
| Sorafenib | 572.4 ± 23.8 | 12.5 ± 3.6 | 0.5 ± 1.7 |
| Regorafenib | 404.7 ± 27.3 | 38.4 ± 4.2* | –5.8 ± 1.4 |
| DBPR114 | 281.7 ± 19.8 | 56.9 ± 3.0*, ** | 1.5 ± 1.5 |

**Table S4. Anti-tumor activity of sorafenib, regorafenib and DBPR114 in sorafenib-acquired resistant human Huh7 HCC xenograft tumors on day 25.** HCC tumors were treated with DBPR114 once a week intravenously for 3 weeks or sorafenib and regorafenib once a day, 5 days per week orally for 25 days. Mean ± SEM, n = 8 mice per group. **p* < 0.05 vs. vehicle control measured by one-way ANOVA and Bonferroni posttest comparison.

| **Treatment** | **Tumor volume** | **Tumor growth inhibition (TGI),**  **% of vehicle control group** | **Body weight change, % of initial weight** |
| --- | --- | --- | --- |
| Vehicle control | 1,531.1 ± 302.2 | - | 8.9 ± 2.1 |
| Sorafenib | 1,004.2 ± 169.5 | 34.4 ± 10.8 | 2.8 ± 3.6 |
| Regorafenib | 265.8 ± 34.8 | 82.7 ± 2.4* | 0.6 ± 2.5 |
| DBPR114 | 178.2 ± 19.3 | 88.4 ± 1.3* | –0.4 ± 2.2 |
